# Based on Bayesian multivariate skewed regression analysis: the interaction between skeletal muscle mass and left ventricular mass

**DOI:** 10.3389/fphys.2025.1515560

**Published:** 2025-08-01

**Authors:** Zhenchao Liu, Tianxiang Lei, Tianwei Liu, Yunliang Guo, Yun Wang, Yu Cao

**Affiliations:** ^1^School of Pharmacy, Qingdao University, Qingdao, China; ^2^Medical Research Center and Cerebrovascular Disease Institute, The Affiliated Hospital of Qingdao University, Qingdao, China; ^3^Health Management Center, Linyi People’s Hospital, Linyi, China; ^4^Clinical Trials Center, The Affiliated Hospital of Qingdao University, Qingdao, China

**Keywords:** sarcopenia, left ventricular mass, Bayesian multivariate skewed regression, skeletal muscle mass, skewness, heavy-tailed characteristics

## Abstract

**Objective:**

This study aims to investigate the association between skeletal muscle mass (SMM) and left ventricular mass (LVM), providing a basis for health management and cardiac health interventions in sarcopenic populations.

**Methods:**

We conducted a retrospective analysis of participants who underwent SMM assessment at Linyi People’s Hospital from January 2017 to December 2023, including a total of 278 individuals. The study employed Bayesian multivariate skewed regression analysis, incorporating ridge regression as a prior distribution to address the skewness and heavy-tailed characteristics of the LVM data. Data collection included clinical information, SMM, and cardiac function metrics. Posterior inference was conducted using Markov Chain Monte Carlo (MCMC) methods, and model convergence was assessed through Gelman-Rubin diagnostics.

**Results:**

The results of ridge regression indicate that age (*β* = 4.54, *95% CI* = 1.23–7.85) and appendicular lean mass (ALM) (*β* = 16.82, *95% CI* = 2.87–30.77) are significantly positively correlated with LVM. In contrast, Bayesian multivariate skewed regression analysis demonstrates that the skeletal muscle index (SMI) (*β* = 22.22, *95% CI* = 2.41–39.07) exerts a significant positive effect on LVM. Additionally, locally weighted scatterplot smoothing (LOWESS) analysis reveals that LVM tends to increase with higher levels of both ALM and SMI.

**Conclusion:**

This study found that skeletal muscle mass (such as ALM and SMI) is significantly associated with LVM, suggesting that there is an association between improvements in skeletal muscle and a potential positive impact on cardiac health, highlighting the importance of regional muscle mass. These findings provide new insights for cardiac health management in sarcopenic populations, indicating that there is a relationship where interventions could potentially involve enhancing ALM.

## 1 Background

Sarcopenia is a syndrome characterized by the progressive loss of skeletal muscle mass (SMM) and strength associated with aging, often accompanied by functional impairment and a decline in quality of life (Narici and Maffulli, 2010). The causes of sarcopenia primarily include age-related declines in muscle synthesis capacity, lack of physical activity, malnutrition (particularly insufficient protein intake), chronic diseases (such as diabetes and heart disease), hormonal changes, and chronic inflammation (Narici and Maffulli, 2010; Liu et al., 2024a; Chandrashekhar et al., 2023). Additionally, degeneration of nervous system function can also affect muscle control and utilization efficiency. Despite the growing understanding of sarcopenia, challenges remain in clinical management and intervention, such as difficulties in early identification, adherence to intervention measures, the need for individualized management, and insufficient multidisciplinary collaboration (Narici and Maffulli, 2010; Kirk et al., 2020). Recent studies have increasingly indicated that low SMM not only affects an individual’s mobility and quality of life but may also be closely related to changes in cardiac function. Current perspectives suggest that the reduction of SMM may be associated with an increased risk of cardiac remodeling and heart failure (Liu et al., 2024a). Left ventricular mass (LVM) is an important indicator of cardiac health, and existing research has shown a correlation between SMM and LVM; however, significant controversy remains regarding their causal relationship. For instance, Keng et al. (2019) conducted a study involving 378 participants aged ≥65 years, finding that 23.3% had sarcopenia. Their multivariate linear regression analysis indicated a significant positive correlation between SMM and LVM. Similarly, Pelà et al. (2020) analyzed 100 participants aged 70 years and older, and their multivariate linear regression analysis also revealed a positive correlation between LVM and appendicular lean mass (ALM), indicating that LVM increases with ALM. Tinti et al. (2022) studied 228 participants aged 65–91 years and found that those with low SMM had lower LVM compared to those with normal muscle mass; partial correlation analysis showed significant correlations between LVM and both ALM and skeletal muscle index (SMI). Wang et al. (2020) observed that among patients with chronic heart failure, those with sarcopenia exhibited lower LVM compared to patients with normal skeletal muscle levels. Their multivariate linear regression analysis demonstrated a significant linear positive correlation between LVM and SMI. Collectively, an increasing body of research indicates a close relationship between sarcopenia and cardiac health, particularly suggesting that the reduction in SMM may significantly correlate with changes in LVM. Liu et al. (2024b) studied patients with hyperthyroidism aged 18 years and older, and the results from generalized linear models and structural equation modeling also indicated correlations between SMM, SMI, and LVM. The integrative mechanisms between cardiac and skeletal muscle quality may include several aspects. First, skeletal muscle, as a major metabolic organ, can directly influence systemic blood circulation and oxygen delivery through changes in its mass and function, thereby affecting the cardiac workload. For instance, the secretion of Akt (protein kinase B) by skeletal muscle is considered a cardioprotective factor that helps to reduce cardiac injury. A decrease in Akt levels within muscle tissue may diminish this protective effect, thereby increasing the risk of cardiac damage (Pelà et al., 2020; Liu Y. et al., 2024). Second, declining cardiac health can lead to inadequate blood oxygenation and reduced physical activity, further exacerbating sarcopenia or the decline in skeletal muscle quality (Keng et al., 2019; Wang et al., 2020). Lastly, both cardiac and skeletal muscle health are influenced by common risk factors such as aging, obesity, and metabolic syndrome, which can create a negative feedback loop between the two. For example, insulin resistance can induce concurrent alterations in both cardiac and skeletal muscle functions; impaired insulin signaling can reduce the efficiency of glucose utilization in cardiomyocytes, leading to insufficient energy production and compromised cardiac function. At the same time, decreased levels of insulin-like growth factor 1 (IGF-1) can negatively affect skeletal muscle protein synthesis, resulting in a decline in muscle quality and accelerating the onset of sarcopenia. This metabolic imbalance not only impacts the health of both the heart and skeletal muscles but may also interact to further exacerbate symptoms of heart failure and muscle loss (Tang et al., 2020; Aghdam et al., 2020; Ungvari and Csiszar, 2012). Overall, there exists a close interrelationship between muscle and cardiac health, and maintaining the wellbeing of both is crucial for the overall health of the elderly population.

Although some cross-sectional studies have revealed a potential link between low SMM and LVM, these studies have notable limitations. First, many of them fail to account for a wider range of potential confounding factors, which could lead to biased results. Second, the traditional statistical methods employed in most studies may not adequately capture the complex relationships between variables, particularly in cases of small sample sizes or weak effect sizes. Finally, most studies focus primarily on populations aged 65 and older, which may restrict the generalizability of the findings and fail to fully represent the relationship between muscle quality and cardiac health across different age groups and backgrounds. To effectively address these issues, the introduction of Bayesian analysis methods is particularly necessary. Bayesian approaches, by integrating prior knowledge with observational data, can provide more reliable inferences in situations of high uncertainty. The advantages of this method lie in its flexibility and adaptability, as it can effectively handle confounding factors and missing data, thereby enhancing the stability and interpretability of the models. Additionally, Bayesian analysis allows researchers to utilize prior research findings as prior information, which not only improves the accuracy of the model but also better reflects the complexity of the real world. By employing Bayesian methods, it becomes possible to delve deeper into the relationship between SMM and LVM, ultimately providing more targeted insights for health management and intervention strategies in sarcopenic populations.

## 2 Study subjects and methods

### 2.1 Study subjects

This retrospective analysis included participants who underwent skeletal muscle evaluation through dual-energy X-ray absorptiometry (DXA) at Linyi People’s Hospital from January 2017 to December 2023. Based on the inclusion and exclusion criteria, a total of 278 participants were enrolled in the study, with ages ranging from 18 to 91 years. Participants were selected based on predetermined inclusion and exclusion criteria. Those with low skeletal muscle mass were included in the study group, while normal participants served as the control group. The studies involving human participants were reviewed and approved by the Science and Technology Ethics Committee of Linyi People’s Hospital (Ethical Review No.: 202404-H-021) and was conducted in accordance with the principles outlined in the Declaration of Helsinki. The use of the data has been approved by the appropriate ethics committee to ensure compliance with ethical standards.

#### 2.1.1 Diagnostic criteri

The diagnostic criteria for low SMM were based on the 2019 criteria from the Asian Working Group for Sarcopenia (Chen et al., 2020): SMI <7.0 kg/m^2^ for males and SMI <5.4 kg/m^2^ for females, assessed using DXA.

#### 2.1.2 Inclusion criteria

(1) Age ≥18 years; (2) Participants who had undergone echocardiographic examination; (3) Availability of complete baseline data, including sex, age, weight, medical history, current medical status, smoking history, and alcohol consumption history.

#### 2.1.3 Exclusion criteria

(1) Participants with severe cardiovascular diseases, such as heart failure, that could lead to changes in cardiac structure; (2) Participants who had undergone surgery, experienced fractures, or had other conditions that could lead to prolonged bed rest and disuse of muscles; (3) Participants who had been on long-term medications such as growth hormones or estrogen that might affect SMM; (4) Based on previous studies, multiple pregnancies may have an impact on cardiac structure; therefore, female participants with more than four pregnancies were excluded (Gunderson et al., 2011); (5) Participants who have engaged in long-term athletic occupations may have altered cardiac structures (Wisløff et al., 2009); (6) Participants involved in prolonged high-load occupations and night shift work may also experience changes in cardiac structure (Trott et al., 2022).

### 2.2 Study methods

#### 2.2.1 Data collection

The objective of this study was to retrospectively investigate the association between skeletal muscle conditions and LVM in individuals with low SMM. Clinical data for all eligible participants were collected as covariates, including age, sex, height, weight, smoking status (defined as smoking more than 20 cigarettes daily for over 10 years, or more than 40 cigarettes daily for over 5 years), alcohol consumption status (defined as drinking alcohol for more than 300 days per year or consuming more than 120 g of alcohol per month), lipid profiles, medical history, current medical conditions, smoking history, drinking history. Body Mass Index (BMI) was calculated as BMI = weight (kg)/height (m^2^).

Based on the results of DXA scans (GE, United States), data were collected on total SMM, upper limb skeletal muscle mass (UpSMM), and lower limb skeletal muscle mass (LowSMM) for all participants. The SMI was calculated as SMI = ALM (kg)/height (m^2^).

In addition, echocardiography (EPIQ7, PHILIPS, Netherlands) was performed to collect data on the left atrium (LA), aorta (AO), left ventricular diastolic diameter (LVDd), interventricular septal thickness at end-diastole (IVSTd), left ventricular posterior wall thickness at end-diastole (LVPWTd), right ventricle (RV), ascending aorta (AAO), main pulmonary artery (MPA), ejection fraction (EF), and fractional shortening (FS). LVM was calculated using the formula (Wang et al., 2018):

$$\text{LVM}=0.8\times 1.04\times \left[{\left(\text{LVDd}+\text{LVPWTd}+\text{IVSTd}\right)}^{3}-{\text{LVDd}}^{3}\right]+0.6$$

#### 2.2.2 Statistical analysis

We first performed normality tests for all variables and selected appropriate statistical methods based on the results. For variables that did not conform to a normal distribution, non-parametric tests or data transformations were employed. Comparisons between the study group and the control group, as well as between subgroups, were conducted using t-tests or Mann-Whitney U tests for continuous data, while categorical data were compared using chi-square tests. The analysis indicated that the LVM (left ventricular mass) data were right-skewed (Skewness: 0.92), suggesting that conventional linear regression models might not be suitable for these data. Additionally, while generalized linear models (GLMs) can handle non-normally distributed data, they typically still require the assumption of a specific distribution form, such as Poisson or binomial, which is not applicable in our study. Therefore, we chose a Bayesian multivariate skew regression model. This model not only naturally accommodates the skewness of the data but also allows us to incorporate existing information through prior distributions, thus enhancing the stability and accuracy of parameter estimates. In this process, we performed ridge regression analysis to address multicollinearity issues. Ridge regression reduces the variance of the regression coefficients by introducing an L2 regularization term, thereby enhancing the model’s stability. However, using ridge regression as prior information may have its limitations. For example, the regularization process of ridge regression can lead to estimated regression coefficients being biased toward zero, which can affect the interpretability of the model. Additionally, ridge regression assumes that the relationships between all explanatory variables are linear, which may not be applicable in all data contexts. Due to these limitations, we chose to use non-informative priors to reduce the model’s dependence on specific prior distributions, thereby enhancing the model’s flexibility and broad applicability. By employing skewed Student’s t-distribution and skewed-I Student’s t-distribution, we effectively addressed the asymmetry and heavy-tailed properties of the data. The Bayesian model incorporated prior distributions of parameters through a prior product structure, which included the distributions of regression coefficients, transformation matrices, and skewness parameters, allowing for varying degrees of freedom and tail behavior, thus providing more comprehensive support for the model’s estimation. For inference, Markov Chain Monte Carlo (MCMC) methods were utilized for posterior inference, ensuring that the model remained flexible and accurate when handling complex data (Tang et al., 2020; Aghdam et al., 2020). Assuming we have n observations represented as (*x*_*i*_, *y*_*i*_), where *x*_*i*_ is the vector of explanatory variables and *y*_*i*_ is the variable of interest, each *y*_*i*_ can be expressed as: $y_{i}=g_{i}\left(B\right)+{\lambda_{i}}^{-1/2}A^{T}\epsilon_{i}$ , where *g*_*i*_ (B) is a known regression function, B represents the regression coefficients, A is the transformation matrix that introduces dependencies among different components, and *ϵ*_*i*_ is the residual under a multivariate standard normal distribution. During the MCMC sampling, we set up 32 chains with 20,000 iterations to ensure model convergence and the reliability of results. Following the MCMC sampling, sensitivity analyses were conducted to assess the robustness of the model against different prior distributions and other assumptions. Following collinearity analysis, we found collinearity issues among SMI, SMM, ALM, and BMI (VIF>10). Therefore, we incorporated an L2 regularization step into the Bayesian analysis process. The introduction of skewness parameters and the treatment of residuals allowed us to address the skewness characteristics of the dependent variable. Finally, we employed locally weighted scatterplot smoothing (LOWESS) to fit the correlation trends between skeletal muscle mass parameters and LVM. All statistical analyses were performed using Python 3.10 (https://www.python.org) and R x64 4.1.0 (https://www.r-project.org), with statistical significance set at *P* < 0.05.

## 3 Results

In this study, 104 participants were aged 60 years and older, including 159 females and 119 males. A total of 80 participants were diagnosed with low SMM, with an age range of 18–91 years.

### 3.1 Comparison of baseline characteristics between the two groups

A comparison of clinical and cardiac measurement variables was conducted between the study group (n = 80) and the control group (n = 198). The analysis revealed no significant differences in age and sex between the two groups (*P* = 0.322 and *P* = 0.121). While there were significant differences in skeletal muscle-related parameters, the BMI of the study group was significantly lower than that of the control group (22.34 kg/m^2^ vs 25.16 kg/m^2^, *P* < 0.001). Other clinical variables showed no significant differences in the incidence of type 2 diabetes mellitus (T2DM), hypertension, malignancies, rheumatic autoimmune diseases, and dyslipidemia between the groups.

Regarding cardiac measurement indices, the results indicated that the measurements for LA, IVSTd, LVPWTd, AAO, and MPA were significantly smaller in the study group compared to the control group. Additionally, FS also showed a significant difference between the two groups, suggesting that the study group may have some degree of cardiac functional protection. Finally, LVM was significantly lower in the study group compared to the control group (118.67 g vs 137.48 g, *P* < 0.001) (see Table 1).

**TABLE 1:** The comparison of variables between the Study group and the control group.

Variable	Study group (n = 80)	Control group (n = 198)	*t*/*z*/*x* ^2^	*P*
Clinical covariates				
Age (years) (med, IQR)	53.50 (31.80)	52.50 (22.30)	0.99	0.322
Age<60years/>60years	45/35	131/67	2.41	0.121
Female (%)	44 (55.00)	115 (58.08)	0.22	0.638
Height (cm) (*x̄*±*s*)	161.62 ± 8.74	164.11 ± 8.99	2.13	0.034
Weight (kg) (med, IQR)	59.50 (14.80)	67.20 (19.90)	5.47	<0.001
BMI (kg/m^2^) (med, IQR)	22.34 (4.37)	25.16 (5.27)	5.58	<0.001
SMI (kg/m^2^) (med, IQR)	5.30 (1.59)	6.79 (1.78)	8.74	<0.001
Male-SMI (kg/m^2^) (med, IQR)	6.55 (0.56)	7.82 (0.77)	8.64	<0.001
Female-SMI (kg/m^2^) (med, IQR)	5.00 (0.54)	6.17 (0.72)	9.74	<0.001
SMM (kg) (med, IQR)	33.77 (10.74)	41.60 (15.25)	6.42	<0.001
UpSMM (kg) (med, IQR)	3.41 (1.50)	4.50 (2.06)	6.76	<0.001
LowSMM (kg) (med, IQR)	10.27 (4.11)	13.00 (5.06)	7.31	<0.001
ALM (kg) (med, IQR)	13.49 (5.92)	17.42 (7.08)	7.42	<0.001
T2DM(%)	18 (22.50)	49 (24.75)	0.16	0.692
Hyperthyroidism (%)	16 (20.00)	23 (11.62)	3.32	0.068
Malignant tumor (%)	5 (6.25)	5 (2.53)	2.28	0.131
Hypertension (%)	11 (13.75)	39 (19.70)	1.37	0.242
Rheumatic immune disease (%)	4 (5.00)	7 (3.53)	0.32	0.571
Dyslipidemia^a^(%)	9 (11.25)	19 (9.60)	0.17	0.678
Smoking (%)	30 (37.50)	73 (36.87)	0.01	0.921
Alcohol consumption (%)	31 (38.75)	85 (42.93)	0.41	0.522
Cardiac measurements				
LA (mm) (med, IQR)	31.00 (3.80)	32.00 (4.00)	2.33	0.020
AO (mm) (med, IQR)	20.00 (2.00)	21.00 (2.0)	1.62	0.106
LVDd (mm) (med, IQR)	45.50 (3.00)	46.00 (4.00)	1.35	0.178
IVSTd (mm) (med, IQR)	8.00 (1.00)	9.00 (2.00)	5.08	<0.001
LVPWTd (mm) (med, IQR)	8.00 (1.00)	9.00 (2.00)	4.33	<0.001
RV (mm) (med, IQR)	20.50 (1.00)	21.00 (2.00)	1.37	0.172
AAO (mm) (med, IQR)	29.50 (4.00)	31.00 (5.00)	3.00	0.003
MPA (mm) (med, IQR)	20.00 (1.00)	21.00 (2.00)	2.33	0.020
EF (%) (med, IQR)	60.00 (2.00)	58.00 (3.00)	1.93	0.053
FS^b^ (%) (med, IQR)	31.00 (2.00)	30.00 (1.30)	2.10	0.036
LVM (g) (med, IQR)	118.67 (25.52)	137.48 (40.40)	4.54	<0.001

SMI, skeletal muscle index; SMM, skeletal muscle mass; UpSMM, up limb skeletal muscle mass; LowSMM, lower limb skeletal muscle mass; BMI, body mass index; LA, left atrial diameter; AO, aortic root diameter; LVDd, Left Ventricular End-Diastolic Diameter; IVSTd, Interventricular Septal Thickness at End-Diastole; LVPWTd, Left Ventricular Posterior Wall Thickness at End-Diastole; RV, right ventricular diameter; AAO, ascending aortic diameter; MPA, main pulmonary artery diameter; EF, ejection fraction; FS, fractional shortening; LVM, left ventricular mass; T2DM, Type 2 Diabetes Mellitus.

^a^ Due to missing specific lipid data such as total cholesterol and triglycerides for 67 participants, with only previous history records available, we only recorded whether blood lipids were abnormal.

^b^ For two participants with missing and unrecorded FS (Fractional Shortening) values, the median value was used to impute the data.

### 3.2 Bayesian multivariate skew regression analysis

Upon analyzing the distribution characteristics of LVM, we observed a skewness of 0.92, indicating a right-skewed distribution of the data. Additionally, the Shapiro-Wilk test yielded a statistic of 0.953 with a *P* = 9.03e^−8^, further supporting the rejection of the normality assumption for the data and indicating that LVM does not conform to a normal distribution. These features could potentially affect the validity and inference of traditional statistical methods. Therefore, to model and analyze this data more accurately, we opted for Bayesian multivariate skew regression. This approach not only accommodates non-normally distributed data but also effectively captures the skewness and heavy-tail characteristics of the data. Its flexibility makes it particularly robust and reliable for analyzing complex biomedical data. By incorporating prior distributions, we were able to leverage existing knowledge and data for inference, thereby enhancing the accuracy and interpretability of the model.

#### 3.2.1 Ridge regression analysis results

In this study, we selected ridge regression, due to its effectiveness in handling multicollinearity and high-dimensional data, as well as its stability in parameter estimation. Following the ridge regression analysis of LVM, the results indicated that age, ALM had significant positive effects on LVM (see Table 2).

**TABLE 2:** Ridge regression results of skeletal muscle mass variables and LVM.

Variable	β	Standard error	*t*	*P*	*95%CI*
Const	135.11	1.45	92.92	<0.001	132.24–137.93
Age	4.54	1.68	2.71	0.007	1.23–7.85
Gender	−0.63	2.51	−0.25	0.803	−5.58–4.32
SMI	5.47	4.82	1.14	0.258	−4.03–14.97
SMM	−8.03	4.69	−1.71	0.088	−17.28–1.21
ALM	16.82	7.08	2.38	0.018	2.87–30.77
BMI	1.78	1.95	0.91	0.362	−2.07–5.63
T2DM	−3.14	1.64	−1.92	0.057	−6.36–0.09
Hyperthyroidism	−0.96	1.63	−0.59	0.556	−4.16–2.25
Malignant tumor	1.14	1.51	0.76	0.451	−1.84–4.12
Hypertension	−0.77	1.54	−0.50	0.617	−3.80–2.26
Rheumatic immune disease	2.87	1.51	1.90	0.059	−0.11–5.85
Dyslipidemia	0.629	1.58	0.40	0.691	−2.48–3.74
Smoking	−3.95	2.59	−1.52	0.129	−9.07–1.16
Alcohol consumption	2.76	2.70	1.02	0.308	−2.56–8.08

SMI, skeletal muscle index; SMM, skeletal muscle mass; UpSMM, up limb skeletal muscle mass; LowSMM, lower limb skeletal muscle mass; BMI, body mass index; LVM, left ventricular mass; T2DM, Type 2 Diabetes Mellitus.

#### 3.2.2 Bayesian multivariate skew regression analysis results

**FIGURE 1:**
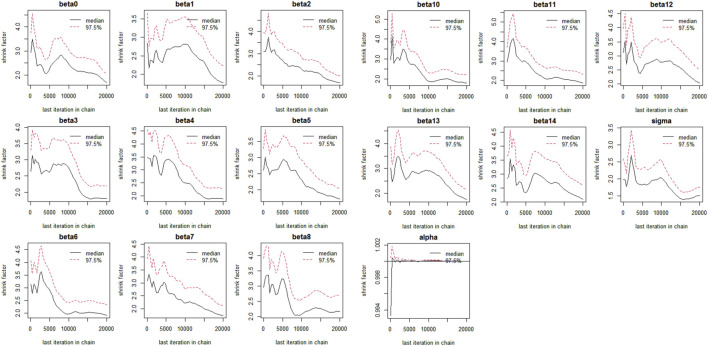
Evaluation of Convergence Diagnostics for Bayesian Regression Model Parameters beta1: Age, beta2: Gender, beta3: SMI, beta4: SMM, beta5: ALM, beta6: Alcoholcon sumption, beta7: BMI, beta8: T2DM, Beta9: Hyperthyroidism, beta10: Malignanttumor, beta11: Hypertension, beta12: Rheumaticimmunedisease, beta13: Dyslipidemia, beta:Smoking.

**FIGURE 2:**
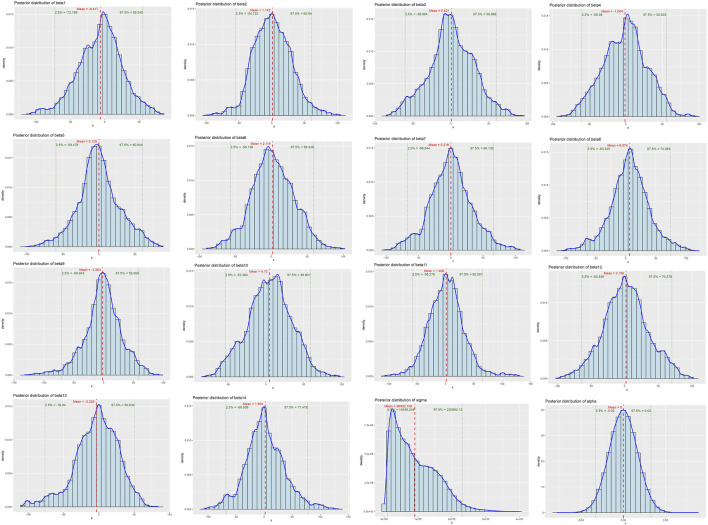
Posterior Distributions of Bayesian Regression Parameters. beta1: Age, beta2: Gender, beta3: SMI, beta4: SMM, beta5: ALM, beta6: Alcoholcon sumption, beta7: BMI, beta8: T2DM, Beta9: Hyperthyroidism, beta10: Malignanttumor, beta11: Hypertension, beta12: Rheumaticimmunedisease, beta13: Dyslipidemia, beta:Smoking.

The Bayesian multivariate skew regression analysis revealed that only SMI exhibited a significant positive association with LVM, suggesting that improvements in muscle mass may have a beneficial impact on cardiac health. Other variables, such as SMM, ALM, and T2DM, did not demonstrate statistical significance, which may reflect the complex interactions among different factors influencing LVM (see Table 3). Most parameter shrinkage factors stabilized around a value close to 2 after sufficient iterations, indicating that these estimates are relatively reliable (see Table 4; Figure 1 Evaluation of Convergence Diagnostics for Bayesian Regression Model Parameters). In this study, the root mean square error (RMSE) was 126.92, and the mean absolute error (MAE) was 23.68, indicating an acceptable level of predictive performance. The posterior distributions of the majority of parameters showed clear concentration, implying stable estimations. For the standard deviation parameter σ, the posterior distribution was skewed towards higher values and relatively broad, indicating substantial residual variability and some uncertainty in the noise estimation (see Figure 2 Posterior Distributions of Bayesian Regression Parameters).

**TABLE 3:** Bayesian Multivariate Skewed Regression results of skeletal muscle mass variables and LVM.

Variable	β(Mean)	Standard error	*95%CI*
Age	−14.78	17.03	−42.28–22.70
Gender	8.89	21.30	−35.73–46.80
SMI	22.22	9.62	2.41–39.07
SMM	−2.41	13.61	−33.33–20.16
ALM	22.20	18.13	−11.45–52.75
BMI	−12.76	11.38	−33.80–9.03
T2DM	−22.96	20.67	−60.32–19.41
Hyperthyroidism	2.42	22.30	−36.47–38.00
Malignant tumor	−2.18	25.92	−52.46–42.89
Hypertension	23.76	18.25	−12.82–55.98
Rheumatic immune disease	−3.93	19.13	−39.85–23.68
Dyslipidemia	20.72	14.36	−7.00–44.80
Smoking	16.37	14.99	−8.86–48.08
Alcohol consumption	3.30	23.29	−48.56–44.89
Sigma	49,143.01	29,059.01	10,286.22–112,265.00
Alpha	0.00004	0.01	−0.02–0.02

SMI, skeletal muscle index; SMM, skeletal muscle mass; UpSMM, up limb skeletal muscle mass; LowSMM, lower limb skeletal muscle mass; BMI, body mass index; LVM, left ventricular mass; T2DM, Type 2 Diabetes Mellitus.

**TABLE 4:** Gelman-rubin diagnostic coefficients for each parameter.

Variable	*R*
Age	1.786
Gender	1.701
SMI	1.808
SMM	1.884
ALM	1.721
BMI	1.920
T2DM	1.748
Hyperthyroidism	2.176
Malignant tumor	2.010
Hypertension	1.818
Rheumatic immune disease	1.880
Dyslipidemia	2.037
Smoking	1.768
Alcohol consumption	2.099
Sigma	1.521
Alpha	1.000

SMI, skeletal muscle index; SMM, skeletal muscle mass; UpSMM, up limb skeletal muscle mass; LowSMM, lower limb skeletal muscle mass; BMI, body mass index; LVM, left ventricular mass; T2DM, Type 2 Diabetes Mellitus.

### 3.3 LOWESS results

**FIGURE 3:**
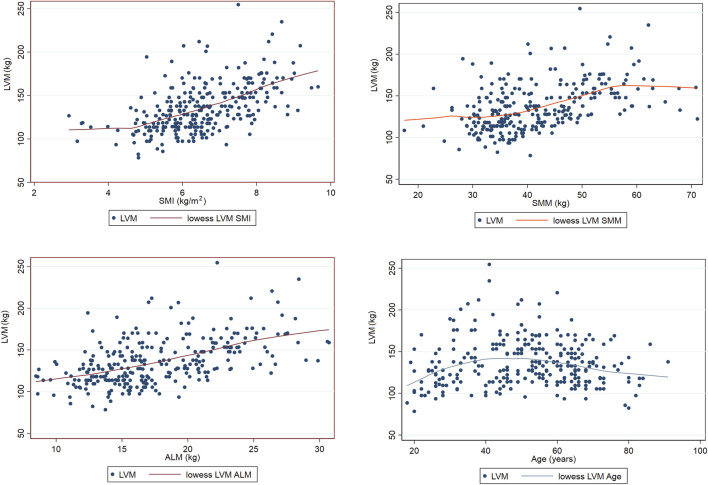
The relationship between left ventricular mass and body composition metrics by LOWESS.

**FIGURE 4:**
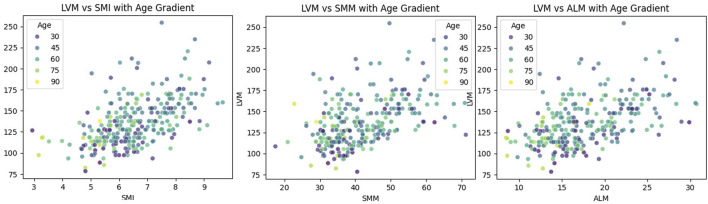
LVM correlation with body composition metrics: Age gradient analysis.

**FIGURE 5:**
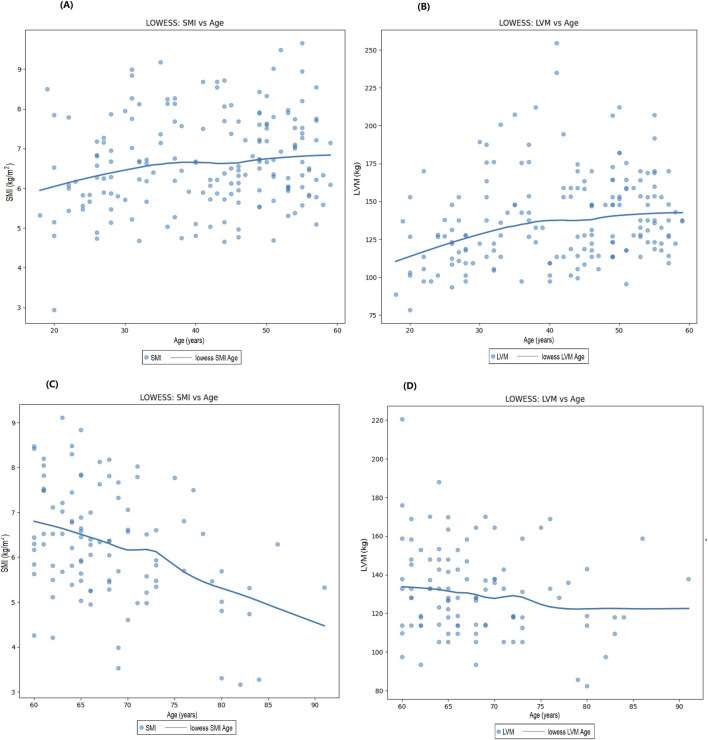
Age-related trends in SMI and LVM by LOWESS. **(A)** Trends in sSMI with age in participants under 60 years, **(B)** Trends in LVM with age in participants under 60 years, **(C)** Trends in SMI with age in participants over 60 years, **(D)** Trends in LVM with age in participants over 60 years.

The LOWESS smoothing curve analysis demonstrated the relationships between LVM and Age, SMI, SMM, and ALM. The results indicated that, overall, as LVM increased, all muscle indicators exhibited a rising trend. We further illustrated the trends and slopes of several muscle parameters in relation to age, as well as the trends of SMI and LVM across different age groups (those older than 60 years and those younger than 60 years). SMI decreased with age in both groups, while LVM exhibited a gradual upward trend with increasing age in participants under 60 years. However, in the population over 60 years, LVM showed more complex variations. (see Figure 3 The Relationship Between Left Ventricular Mass and Body Composition Metrics by LOWESS; Figure Figure4 LVM Correlation with Body Composition Metrics: Age Gradient Analysis; Figure Figure5 Age-Related Trends in SMI and LVM by LOWESS).

## 4 Discussion

Previous studies primarily employed linear regression analysis (Keng et al., 2019; Pelà et al., 2020; Tinti et al., 2022). However, the relationship between muscle quality indicators (such as ALM and SMI) and cardiac structure indicators (such as LVM) may be influenced by the correlations among independent variables. This can lead to instability and unreliability in the coefficient estimates of classical linear regression, particularly when there is high multicollinearity among the independent variables, which results in larger variance and reduced predictive performance of the model (Alin, 2010). Additionally, linear regression models often demonstrate certain limitations when facing skewed distribution data, particularly when the data do not conform to normality assumptions, which can lead to misinterpretation of the true relationships, especially when addressing complex variables. Ridge regression is a commonly used extension of linear regression designed to address multicollinearity issues. By incorporating an L2 regularization term, ridge regression effectively shrinks the regression coefficients, thereby mitigating the impact of multicollinearity. This shrinkage enhances the stability of the model in the presence of multicollinearity and provides more reliable coefficient estimates with lower prediction error (Schreiber-Gregory, 2018). In our study, ridge regression results indicated a positive correlation between ALM and LVM, which is consistent with previous studies (Keng et al., 2019; Pelà et al., 2020; Tinti et al., 2022), supporting the notion of a strong relationship between skeletal muscle quality and cardiac health. This finding suggests that maintaining higher limb muscle quality may positively influence cardiac structural characteristics. Despite its advantages in managing strong correlations among independent variables, ridge regression does not require data to strictly adhere to normal distribution assumptions. However, due to the introduction of the L2 regularization term, ridge regression may shrink the regression coefficients, potentially underestimating the actual effects of certain variables, especially in cases where relationships among variables are complex. Furthermore, ridge regression does not perform variable selection, which may increase model complexity and hinder interpretability (Wu, 2021). Consequently, we further introduced Bayesian Multivariate Skewed Regression Analysis in this study, which not only better accommodates the skewed nature of the data but also effectively addresses the impact of heavy-tailed distributions. Through Bayesian Multivariate Skewed Regression Analysis, we observed that the relationship between SMI and LVM was significantly stronger than that between ALM and LVM. This finding suggests that while ALM may be related to LVM to some extent, SMI, as a more comprehensive measure of muscle quality, more effectively reflects its close association with cardiac health. Moreover, compared to earlier studies, our sample encompassed a broader age range (18–91 years), enhancing the generalizability and clinical relevance of the results, and enabling us to better assess the impact of muscle quality on cardiac health.

When assessing an individual’s muscle quality, the SMI is widely recognized as a significant biomarker, particularly in the diagnosis and study of sarcopenia, as it effectively reflects the level of skeletal muscle quality in individuals (Liu et al., 2024b). This study employed ridge regression analysis to explore the relationship between SMI and LVM, revealing no significant correlation between the two. However, the use of LOWESS analysis indicated a discernible upward trend between SMI and LVM, while Bayesian Multivariate Skewed Regression further demonstrated a positive correlation between SMI and LVM. The discrepancies observed between the ridge regression and Bayesian multivariate skewed regression results may stem from the differing assumptions and model structures inherent to these statistical methods. As previously mentioned, ridge regression aims to address multicollinearity issues by introducing regularization techniques to enhance model stability and interpretability, whereas Bayesian multivariate skewed regression considers the distribution characteristics of the data during modeling, thereby more effectively capturing nonlinear relationships and complex interactions. Consequently, the Bayesian approach can provide a more nuanced understanding of the potential correlations between SMI and LVM. These corroborative results suggest that under specific physiological or pathological conditions, the impact of SMI on LVM may indeed exist.

When exploring the potential mechanisms linking SMI and LVM, it is pertinent to consider the regulatory role of various physiological factors. With advancing age, the elevation of chronic inflammation and oxidative stress levels is recognized as a critical factor affecting muscle quality and cardiac health (Angelaki et al., 2021; Loh et al., 2022). Specifically, increased levels of inflammatory markers such as interleukin-1β (IL-1β) and tumor necrosis factor-alpha (TNF-α) not only activate multiple intracellular signaling pathways that disrupt normal muscle metabolism and promote muscle protein degradation, ultimately leading to muscle atrophy (Teixeira et al., 2021; Meng and Yu, 2010), but also inflict damage on cardiomyocytes, thereby impacting cardiac structure and function, potentially leading to cardiac remodeling and dysfunction (Chang et al., 2023; Wu et al., 2023). Moreover, oxidative stress plays a vital role in this process. As individuals age, the increase in Reactive Oxygen Species (ROS) and the decline in antioxidant defense capacity further exacerbate oxidative stress levels (Loh et al., 2022; Shen et al., 2015). Oxidative stress contributes not only to cellular membrane damage and mitochondrial dysfunction but also promotes apoptosis and autophagy dysregulation in muscle cells by influencing key signaling pathways such as AMPK and mTOR (Wang et al., 2015). The intricate interplay between oxidative stress and inflammation may lead to declines in muscle quality and deterioration of cardiac function, creating a vicious cycle that can impact the relationship between SMI and LVM. In summary, the interaction between SMI and LVM may be jointly influenced by a multitude of physiological and pathological factors, which potentially modulate the relationship between muscle quality and cardiac structure under varying conditions.

Studies by Pelà et al. (2020) and Tinti et al. (2022) have reported significant associations between limb SMM and LVM, suggesting that skeletal muscle quality in the limbs may play a crucial role in maintaining cardiac function. Compared to previous research, this study encompasses a broader sample range, including individuals aged 18–91 years, which enhances the generalizability and applicability of the findings. The metabolic activity of muscle is closely linked to vascular relaxation, and metabolically active load-bearing skeletal muscle enhances the synthesis of nitric oxide (NO) catalyzed by neuronal nitric oxide synthase (nNOS) in muscle cells and endothelial nNOS through the adenosine monophosphate-activated protein kinase (AMPK) signaling pathway. Moreover, post-exercise, both insulin and AMPK can activate endothelial nitric oxide synthase (eNOS) in vascular cells, further promoting nNOS activation and regulating metabolism. Thus, lower limb muscle training can, to some extent, improve cardiovascular function (Linke et al., 2001; Clark et al., 2003; Umbrello et al., 2013; Jie et al., 2009). Sasaki et al. (2017) developed a cardiac cycle-synchronized electrical stimulation device for the lower limbs, which highlighted the significant role of lower limb muscle in alleviating cardiac workload. This device facilitates the flow of hypoxic blood toward the heart and promotes capillary dilation and filling, thereby enhancing the heart’s pumping capacity. This indicates that lower limb muscles may assist with cardiac pumping. Additionally, research by Parissis et al. (2015) demonstrated that lower limb electrical stimulation therapy, which induces muscle contractions, could effectively improve flow-mediated dilation in patients with chronic heart failure. These findings together suggest that limb muscle quality may have a significant impact on cardiac structure; however, existing studies have insufficiently addressed the role of upper limb muscles. This underscores the need for further exploration of the potential effects of upper limb musculature on cardiac function and structure in future research.

In this study, ridge regression and Bayesian multivariate skew regression yielded differing results, indicating that ALM and SMI hold distinct positions as correlating factors. This discrepancy may arise from the differing approaches these methods take in addressing multicollinearity and assumptions regarding data distribution. To address this discrepancy, future research should consider increasing sample size and employing techniques such as cross-validation to assess the robustness of the models, thereby ensuring the reliability of the results. Additionally, conducting multicenter large-scale studies will be beneficial in validating the actual associations of ALM and SMI across diverse populations and exploring their combined effects on cardiac function and muscle health. Furthermore, developing appropriate statistical analysis strategies tailored to different data characteristics will provide valuable guidance for future investigations.

Age is a significant factor influencing sarcopenia, the decline in skeletal muscle mass is closely associated with aging (Liu et al., 2024b). The aging process leads to the depletion of skeletal muscle cells, DNA damage, endoplasmic reticulum stress, mitochondrial dysfunction, contractile impairment, hypertrophic growth, and the development of an age-related secretory phenotype in cardiomyocytes. These changes collectively contribute to detrimental alterations in cardiac structure and function, potentially leading to heart failure and other cardiovascular diseases. These alterations can provoke negative changes in cardiac anatomy and function, ultimately contributing to cardiac aging, dysfunction, and heart failure, while increasing the risk of cardiovascular disease incidence and progression (Tang et al., 2020; Parissis et al., 2015). Cardiomyocytes, which comprise 20%–25% of the total number of cardiac cells, account for 76% of the overall volume of the heart. Most cardiomyocytes quickly lose their proliferative capacity after birth, and with advancing age, these cells undergo hypertrophic growth (Chakravarty et al., 2021; Zeitz and Smyth, 2020). Hypertrophic growth serves as an adaptive mechanism in the adult heart; however, when cardiomyocytes are damaged or lost, the heart undergoes remodeling characterized by hypertrophy of both the cardiomyocytes and the ventricular walls, known as cardiac hypertrophy. This hypertrophy can enhance the heart’s contractile force to cope with increased workload. Thus, the depletion of cardiomyocytes is considered a potential precursor to adaptive hypertrophy, yet this change may ultimately lead to the onset of heart failure (Angelaki et al., 2021; Loh et al., 2022; Chakravarty et al., 2021; Zeitz and Smyth, 2020; Pangonyte et al., 2008). In summary, the potential mechanisms underlying the significant positive impact of age on LVM include the depletion of cardiomyocytes and hypertrophic growth, which may ultimately result in adverse changes in cardiac structure and function. In this study, the LOWESS method was employed to analyze the SMI in participants aged 60 and above, revealing a significant decrease that aligns with existing literature indicating the widespread prevalence of sarcopenia in the elderly population, with risk exacerbating with advancing age (Larsson et al., 2019). Sarcopenia not only affects an individual’s mobility and quality of life but may also have detrimental effects on cardiac health. The reduction in muscle mass can lead to decreased cardiac output and increased cardiac workload, thereby exacerbating structural and functional changes in the heart (von Haehling, 2018). Interestingly, in participants under 60 years of age, the trend in SMI changes was not significant, suggesting that skeletal muscle mass tends to remain relatively stable in this age group. This phenomenon may be attributed to higher metabolic function and hormone levels and levels of physical activity among younger individuals (Cruz-Jentoft et al., 2019; Mitchell et al., 2012). The present study conducted a comparative analysis of cardiac and muscle health between participants aged below and above 60, while exploring the impact of age on LVM. The findings indicated that, in participants under 60, changes in left ventricular mass with advancing age were not significant, whereas, in those aged 60 and above, a rising trend in left ventricular mass was observed. Research by Gardin et al. (Gardin et al., 1997) also indicated a lack of significant association between age and LVM in individuals aged 65 and older. In participants under 60, the increase in left ventricular mass may reflect a favorable adaptation of the heart to physical demands during the growth and development phase, during which the heart typically responds effectively to heightened bodily requirements, promoting myocardial growth and development (Agbaje, 2023; Urbina et al., 1995). However, upon reaching the age of 60 and beyond, changes in left ventricular mass may be closely associated with various factors, including cardiovascular diseases, hypertension, alterations in cardiac structure, and declines in metabolic function (Nakou et al., 2016). Therefore, understanding the interplay among these factors is crucial for developing effective intervention strategies aimed at improving cardiac and muscle health in the elderly population. These changes not only affect muscle function but may also have profound implications for cardiac health.

Despite the absence of significant correlations, the associations of smoking and alcohol history with left ventricular mass (LVM) should not be overlooked. It is well established that smoking causes vascular constriction, which may increase the workload on the heart. Previous studies have demonstrated that even in relatively young populations, smoking adversely affects left ventricular structure, with smokers exhibiting significantly higher LVM compared to non-smokers (Park et al., 2021; Markus et al., 2013). Furthermore, chronic alcohol consumption has also been associated with an increased burden of LVM (Yang et al., 2017). Additionally, a higher body mass index (BMI) is often linked to obesity, which can trigger a range of metabolic issues and cardiovascular diseases, potentially leading to an increase in LVM (Heiskanen et al., 2021). Physical activity is another important factor influencing heart function and structure, and it is closely related to sarcopenia (Zytnick et al., 2021). However, due to the insufficient collection of participants’ daily physical activity information in this study, this aspect remains unaddressed. Thus, the role of these factors in the correlation between skeletal muscle and LVM warrants further investigation.

In addition to hyperthyroidism and type 2 diabetes mellitus (T2DM), which have been confirmed to be associated with secondary sarcopenia (Liu et al., 2024b; Zou et al., 2024), other conditions such as cirrhosis are also commonly regarded as triggers for muscle wasting (Zúñiga González and Roco-Videla, 2023). However, as our study failed to collect complete data on patients with cirrhosis, we are unable to effectively assess and validate this association. Moreover, the relatively small number of participants diagnosed with tumors and autoimmune diseases in this study also somewhat limits the generalizability and representativeness of the findings, potentially impacting the final assessment results. Additionally, some long-term medications, such as metformin, are known to modulate the physiological functions of muscle and heart (Zhu et al., 2023; Zullo et al., 2017) and may play a significant role in the interaction between these two systems. However, the usage of these medications was not systematically recorded and analyzed in our study, preventing us from evaluating their potential influence on the study outcomes.

In this study, we observed that the FS in the low muscle mass group was significantly higher than that in the normal skeletal muscle mass group. We hypothesize that this elevation in FS within the low muscle mass group may be attributed to potential compensatory mechanisms; however, the specific mechanisms underlying this phenomenon warrant further investigation. Due to limitations in manuscript length, we are unable to elaborate on these discussions in the current study. Therefore, we plan to conduct further research to explore the relationship between low muscle mass and left ventricular function, including an analysis of potential influencing factors and underlying mechanisms, with the aim of providing a more comprehensive understanding of cardiac health.

This study employed a retrospective design, which may lead to selection bias and information bias, potentially affecting the reliability of the results. Furthermore, the research was conducted at a single center, which may limit the generalizability of the findings to other healthcare facilities or populations. The minimum age of participants was 18 years, which may further restrict the applicability of the results across different age groups, particularly regarding the elderly population.

In this study, considering the skewed distribution and heavy-tailed characteristics of the data, we employed Bayesian multivariate skew regression analysis for data analysis. This method is capable of flexibly handling non-normally distributed data by incorporating skewness and heavy-tailed distributions, thus allowing for a more effective approach to asymmetry and thick-tail characteristics in multivariate regression analysis. It not only establishes asymmetric relationships between the response variable and predictor variables but also effectively captures the skewness of the data and the dependencies among components. Compared to traditional models, Bayesian multivariate skew regression offers a more comprehensive data modeling capability, thereby enhancing the credibility of the results. Additionally, the Bayesian approach allows for the quantification of uncertainty in parameter estimates, enabling researchers to gain a more holistic understanding of the reliability of the results and to consider uncertainty in the decision-making process, providing a more robust solution for small sample sizes and missing data scenarios (Ferreira and Steel, 2004). Through these advantages, our study is better able to accurately reflect the relationship between limb muscle quality and cardiac structure. However, despite the aforementioned advantages of Bayesian multivariate skew regression, this study does have some limitations. First, due to its retrospective design, there may be selection bias and information bias. Second, although we included 278 participants, the sample size remains limited, which may affect the robustness of the results. Furthermore, while we have made efforts to control for potential confounding factors, unidentified factors may still influence the outcomes. Lastly, in this study, the root mean square error (RMSE) and mean absolute error (MAE) of the Bayesian multivariate skewed regression model indicate that there are still some predictive errors within the model. Therefore, this linear trend suggests that we need to further improve the model, for example, by considering the inclusion of additional variables or more complex nonlinear relationships to enhance the predictive performance and overall accuracy of the model. This finding provides potential directions for optimizing the model in future research. Considering the limitations imposed by the same dataset and sample size, we did not opt to incorporate prior distributions and did not use ridge regression as prior information, but rather conducted a comparative analysis. To address these limitations, future research should consider adopting a prospective study design to better control for confounding factors and reduce biases. Moreover, multi-center studies and larger sample sizes would enhance the generalizability and robustness of the findings. Long-term follow-up studies are also recommended to observe the dynamic interactions between skeletal muscle quality and LVM over time. Additionally, further investigation into the impact of upper limb musculature on cardiac function and structure is warranted to provide a more comprehensive understanding of these relationships.

## 5 Conclusion

In this study, we employed ridge regression and Bayesian multivariate skewed regression analyses. The results indicated that ALM has a significant positive association with LVM, while SMI also exhibited a notable association. These findings suggest an association between improved muscle mass, particularly in the limbs, and a potential beneficial effect on cardiovascular health. This evidence provides important theoretical support for the health management and intervention strategies for elderly patients with sarcopenia, particularly concerning the maintenance and enhancement of heart health. Future research should focus on prospective, multicenter studies to further validate the causal relationship between skeletal muscle quality and left ventricular mass, as well as to explore other potential influencing factors, in order to gain a more comprehensive understanding of the impact of muscle quality on cardiovascular health.

## Data Availability

The raw data supporting the conclusions of this article will be made available by the authors, without undue reservation.
